# Factors Influencing Occupational Grief Among Pediatric Emergency and Intensive Care Unit Nurses in South China: A Cross‐Sectional Study

**DOI:** 10.1155/jonm/5685892

**Published:** 2026-04-02

**Authors:** Yongqi Liang, Xiaoke Jiang, Yue Peng, Li Sun, Hui Liu, Jinlu Chen, Yan Zhou, Yi Luo, Xuan Shi, Caixin Yin

**Affiliations:** ^1^ Women’s and Children’s Medical Center of Guangzhou Medical University, Guangzhou, China; ^2^ School of Nursing, Guangzhou Medical University, Guangzhou, China, gzhmc.edu.cn; ^3^ School of Nursing, Guangdong Pharmaceutical University, Guangzhou, China, gdpu.edu.cn; ^4^ Department of Nursing Administration, Women’s and Children’s Medical Center of Guangzhou Medical University, Guangzhou, China

## Abstract

**Aim:**

This study aimed to assess the level of occupational grief among pediatric emergency and intensive care unit nurses and identify the factors influencing it.

**Design:**

Descriptive cross‐sectional study.

**Methods:**

Conducted from March to May 2024, the study included 265 pediatric nurses from 13 tertiary hospitals in South China. Data were collected via electronic questionnaires, which encompassed sociodemographic questions, the Chinese Version of the Grief Traits and State Scale for Nurses (GSSNs), the Disenfranchised Grief Scale (DGS), the Chinese Version of the Professional Grief Support Scale for ICU Nurses (PGSS‐INs), the Chinese Version of the Psychological Detachment Subscale, and the Chinese Version of the Psychological Resilience Scale. Descriptive statistics, *t*‐tests, one‐way ANOVA, Pearson correlation analysis, and multiple linear regression models were used.

**Results:**

Occupational grief was found to be at a high level among the 265 pediatric nurses (mean score: 48.54 ± 9.458 and range: 17–85). Univariate analysis showed significant differences in grief scores based on age, religion, work experience, death education, end‐of‐life care education, and experienced the loss of a close friend or family member. Pearson correlation analysis found negative correlations between total occupational grief and psychological resilience, psychological detachment, competence in handling patient deaths, acceptance of patient deaths, and psychological preparedness for patient deaths and a positive correlation with disenfranchised grief. Multiple linear regression identified elderly age, lack of death education, personnel loss experience, higher disenfranchised grief, and lower acceptance of patient death as significant factors.

**Conclusion:**

Occupational grief in pediatric emergency and ICU nurses directly impacts mental health. Nursing managers should prioritize mental health, provide systematic death education, and establish psychological support interventions.

**Summary**
• Impact
◦ Occupational grief among pediatric emergency and ICU nurses affects mental health. This study identifies factors like age, death education, and disenfranchised grief. It highlights the need for targeted interventions to support nurse well‐being.
• Reporting method
◦ We have followed the STROBE checklist in reporting this study.
• Patient or public contribution
◦ No patient or public contribution.

## 1. Introduction

Despite advancements in technology and medicine, child mortality remains a significant global concern [1]. The majority of pediatric deaths occur in emergency departments and intensive care units (ICUs) [1, 2]. Pediatric nurses, who develop close therapeutic relationships with their patients, are particularly vulnerable to the emotional impact of these losses. Their repeated exposure to patient death and suffering, combined with the responsibility of supporting grieving families, places them at high risk for profound psychological distress [3].

This phenomenon is primarily defined as “occupational grief,” a distinctive form of sorrow experienced by healthcare professionals following the loss of patients under their care [4]. Common manifestations include sadness, helplessness, sleep disturbances, and difficulty concentrating [5]. The distinctiveness of this grief lies in its tendency to be compounded by what is termed “disenfranchised grief”—a situation in which nurses’ personal sense of loss lacks social validation or appropriate outlets for expression, largely because the caregiver–patient relationship is not accorded the same societal recognition as bonds among kin or friends [6]. The absence of social recognition forces nurses to suppress their grief, preventing its natural resolution [7]. Consequently, this leads to the accumulation of unresolved negative emotions, which may evolve into chronic conditions. These unresolved issues can further manifest as severe outcomes including professional burnout, empathy fatigue, and complicated grief. Ultimately, such developments detrimentally impact both the psychological and physical well‐being of affected individuals, while simultaneously undermining their professional efficacy [8, 9].

The emotional toll is especially high in pediatric emergency departments and ICUs [10–12]. Nurses in pediatric emergency departments and ICUs are routinely exposed to tragic circumstances surrounding a child’s death. Studies indicate that 20% of pediatric ICU nurses experience high levels of secondary traumatic stress following a child’s death [13], and a majority of healthcare workers report needing substantial psychological resilience after such events [14].

While the burden of occupational grief is recognized globally, most existing research originates from Western contexts (Europe and North America), with relatively little in non‐Western regions [4]. In China, however, pediatric nurses face a distinct set of challenges—including relatively low societal recognition, heavy workloads, and complex nurse–patient relationships [15]—which may both marginalize their grief and hinder the development of effective support systems. Furthermore, while previous qualitative studies have provided valuable insights into nurses’ lived experiences with child death [16, 17], a significant gap remains in the quantitative assessment of this phenomenon. To our knowledge, no comprehensive cross‐sectional study has been conducted to quantify the level and influencing factors of occupational grief specifically among pediatric emergency and ICU nurses in China.

Evidence suggests that grief support and psychological detachment can alleviate healthcare workers’ sadness after patient deaths and improve their coping mechanisms [18, 19]. Therefore, this study aimed to examine the current situation and influencing factors of occupational grief among pediatric emergency and intensive care nurses in South China, providing a theoretical foundation for targeted interventions by healthcare managers.

## 2. Methods

### 2.1. Design

This study employs a cross‐sectional design.

### 2.2. Study Population

Based on the principle that the sample size should be 5–10 times the number of independent variables [20], and given 22 independent variables in this study, the initial sample size was calculated as 110–220 (i.e., 22 × 5–22 × 10). The independent variables included 14 items from a general information questionnaire, one dimension from the Chinese Version of the Disenfranchised Grief Scale (DGS), five dimensions from the Chinese Version of the Professional Grief Support Scale for ICU Nurses (PGSS‐INs), one dimension from the Chinese Version of the Psychological Detachment Subscale, and one dimension from the Chinese Version of the Psychological Resilience Scale. After accounting for a potential 20% rate of invalid responses, the target sample size was adjusted to 132–264. A convenience sample of 265 nurses was recruited from pediatric emergency and ICU departments across 13 tertiary hospitals in Guangdong Province, China, between March and May 2024. Inclusion criteria were as follows: (1) nurses currently working on the frontline in pediatric emergency departments or pediatric ICUs; (2) nurses with ≥ 6 months of experience in these departments who have encountered patient deaths during their tenure; (3) nurses with valid qualification certificates; and (4) those who provided informed consent and voluntarily participated in the study. Exclusion criteria were as follows: (1) Nurses who were on leave (e.g., for training, medical, or personal reasons) during the data collection period. (2) Those who did not complete the questionnaire in its entirety.

The study was approved by the Ethics Committee of Guangzhou Women and Children’s Medical Center, Guangzhou Medical University (Ethics Approval Number: [2023] No. 342A01). Permission to distribute the questionnaires was obtained from the Pediatric Emergency and Intensive Care Unit Departments. All potential participants received detailed written information regarding the study aims, procedures, potential benefits, and risks, along with assurance of their right to withdraw at any time without consequence. After having sufficient time to review this information and ask questions, individuals who agreed to participate provided written informed consent before commencing the survey. The collected data were made available solely to the research team for analysis.

### 2.3. Instruments

#### 2.3.1. Chinese Version Sociodemographic Questions

This self‐reported questionnaire includes sociodemographic information (gender, age, educational level, religious beliefs, years of service in the unit, education on death, family residence, civil status, education on terminal period, and grief and experience of losing a closely related family member or friend) and occupational grief‐related factors, such as the frequency of patient deaths encountered, competence in dealing with patient deaths, acceptance of patient deaths, and psychological preparedness for patient deaths. Competence in dealing with patient deaths, acceptance of patient deaths, and psychological preparedness for patient deaths are assessed using single‐item measures on a 5‐point Likert scale, ranging from “Very Incompetent/Very Unaccepting/None” (1 point) to “Very Competent/Very Accepting/Very Adequate” (5 points).

#### 2.3.2. Chinese Version of the Grief Traits and State Scale for Nurses (GSSNs)

The GSSNs, originally developed by Betriana et al. [21], assesses the levels of occupational grief among nursing personnel. The “ grief traits” and “grief state” subscales have Cronbach’s *α* coefficients of 0.84 and 0.86, respectively. In 2023, Wang et al. translated and culturally adapted the Chinese version [22], achieving a Cronbach’s *α* coefficient of 0.813. The Chinese Version of the GSSN includes two subscales, four dimensions, and a total of 17 items. The “grief traits” subscale evaluates personal traits, including emotional fluctuations (4 items) and discomfort with death (3 items), comprising a total of 7 items across two dimensions. The “grief state” subscale assesses responses to death‐related events, including sadness after patient death (5 items) and emotional exhaustion from nursing (5 items), totaling 10 items across two dimensions. All items are scored on a 5‐point Likert scale, ranging from 1 to 5. Scoring for the “grief traits” subscale is as follows: Normal (≤ 18.84), High (18.85–23.87), and Severe (> 23.87). Scoring for the “grief state” subscale is as follows: Normal (≤ 30.73), High (30.74–37.22), and Severe (> 37.22). The combined score of the two subscales is as follows: Normal (≤ 48.45), High (48.46–58.85), and Severe (> 58.85). In this study, the Cronbach’s *α* coefficient for the scale is 0.890.

#### 2.3.3. Chinese Version of the DGS

The DGS was developed by the Chinese scholar Feng et al. [23]. The 7‐item scale uses a 5‐point Likert scale, with responses ranging from “*strongly disagree*” (1 point) to “*strongly agree*” (5 points). Higher total scores indicate greater disenfranchised grief experienced by the participant. In previous studies, the DGS has shown a Cronbach’s alpha of 0.827 [23]. In this study, the Cronbach’s *α* coefficient for the scale is 0.872.

#### 2.3.4. Chinese Version of the PGSS‐INs

The Chinese Version of the PGSS‐INs was developed by Chinese scholar Zhang et al. [24]. The scale comprises five dimensions and 27 items: colleague support (8 items), leadership support (8 items), family support (4 items), friend support (4 items), and organizational support (3 items). It employs a 5‐point Likert scale, ranging from 1 (“*strongly disagree*”) to 5 (“*strongly agree*”). Higher scores indicate a stronger level of occupational grief support received. The scale has been used with ICU nurses and nursing master’s students in China. As reported in previous studies, the PGSS‐INs achieved a Cronbach’s alpha of 0.946 [23]. In this study, the Cronbach’s *α* coefficient for the scale is 0.958, demonstrating high reliability and validity.

#### 2.3.5. Chinese Version of the Psychological Detachment Subscale

This study utilizes the Recovery Experience Questionnaire (REQ)—Psychological Detachment Subscale—to assess individuals’ levels of psychological detachment. The Psychological Detachment Subscale, a unidimensional scale from the REQ developed by Sonnentag and Fritz [25] in 2007, has been used to evaluate psychological detachment in nurses, with a reported Cronbach’s *α* coefficient of 0.84. In 2018, Chinese scholars Lu et al. [26] tested the reliability of the subscale among Chinese nurses, reporting a Cronbach’s *α* coefficient of 0.833. The subscale consists of four items, rated on a 5‐point Likert scale ranging from 1 (“*completely disagree*”) to 5 (“*completely agree*”). Total scores range from 4 to 20, with higher scores reflecting greater psychological detachment. In this study, the Cronbach’s *α* coefficient for the scale is 0.845.

#### 2.3.6. Chinese Version of the Psychological Resilience Scale

This study employed the Chinese version of the Psychological Resilience Scale to assess individuals′ psychological resilience. The original scale, developed by Campbell‐Sills et al. [27] in 2007, was based on Connor’s 2003 Connor–Davidson Resilience Scale (CD‐RISC) [28]. The 10‐item version (CD‐RISC‐10) is a unidimensional scale designed to assess psychological resilience among undergraduate students, with a Cronbach’s *α* coefficient of 0.850. The Chinese version was translated and cross‐culturally adapted by Ye et al. [29] in 2016 to assess the psychological resilience of nursing interns, reporting a Cronbach’s *α* coefficient of 0.851. The scale consists of 10 items, rated on a 5‐point Likert scale ranging from “*never*” to “*always*,” with scores ranging from 0 to 4 points. The total score, calculated as the sum of the 10 items, reflects greater psychological resilience with higher scores. In this study, the scale demonstrated a Cronbach’s *α* coefficient of 0.944, indicating high reliability.

### 2.4. Data Collection

This survey is an electronic questionnaire distributed through the WeChat platform, establishing contact with 13 tertiary hospitals in Guangdong Province, China. The survey provided clear explanations of the research purpose and instructions for completion. Participants could proceed with the questionnaire only after providing informed consent. The final dataset was stored securely and was accessible only to the research team for analysis. Questionnaires with uniform responses to all items were excluded to ensure data reliability. Ultimately, a total of 280 questionnaires were collected, of which 265 were valid, yielding an effective response rate of approximately 94.64%.

### 2.5. Data Analysis

Statistical analysis was performed using SPSS Version 22.0. Descriptive statistics (frequencies, percentages, means, and standard deviations) were used to summarize nurses’ demographic characteristics and the scores of all study instruments, including occupational grief (measured by the GSSN), disenfranchised grief, occupational grief support, psychological resilience, and psychological detachment. Differences in GSSN scores based on demographic characteristics were analyzed using *t*‐tests and one‐way ANOVA. Pearson correlation was employed to examine relationships between occupational grief and psychological resilience, psychological detachment, disenfranchised grief, and occupational grief support. Multivariate linear regression analysis was employed to identify the factors influencing occupational grief among nurses working in acute critical care settings. A *p* value of < 0.05 was considered statistically significant.

## 3. Results

### 3.1. Participants’ Sociodemographic Characteristics

The sample comprised 96.98% female nurses, with 56.61% of them being under 31 years old (see Table 1). Among the participants, 92.83% held bachelor’s degrees, 49.06% were married, and 30.57% had more than 10 years of experience in Pediatric Emergency and Intensive Care Unit. Notably, 60.75% had not attended any courses on death education, and 36.98% had not attended courses on the terminal phase and grief process. In our sample, 71.32% of the respondents reported having lost their closest family members or friends. The proportion of pediatric nurses who had experienced 1–5 pediatric patient deaths was 83.78%. The mean scores for key grief‐related variables were as follows: competence in dealing with patient deaths (3.08 ± 0.752), acceptance of patient deaths (3.30 ± 0.748), and psychological preparedness for patient deaths (3.13 ± 0.789).

**TABLE 1:** Participants’ sociodemographic characteristics.

Variable	*n* (%)
Gender	
Male	8 (3.02)
Female	257 (96.98)
Age	
< 31	150 (56.61)
31∼40	89 (33.58)
> 40	26 (9.81)
Level of education	
College and below	9 (3.40)
Bachelor’s degree	246 (92.83)
Graduate degree	10 (3.77)
Religious belief	
Yes	21 (7.92)
No	244 (92.08)
Years of service in the unit	
≤ 3 years	90 (33.96)
4∼10 years	94 (35.47)
> 10 years	81 (30.57)
Education on death	
Yes	104 (39.25)
No	161 (60.75)
Family residence	
Countryside	128 (48.30)
Town	52 (19.62)
City	85 (32.08)
Civil status	
Married	130 (49.06)
Single	132 (49.81)
Divorced	3 (1.13)
Education on terminal period and grief	
Yes	167 (63.02)
No	98 (36.98)
Loss of close family member or friend	
Yes	189 (71.32)
No	76 (28.68)
Number of patient deaths experienced	
1∼5	222 (83.78)
6∼10	10 (3.77)
> 10	33 (12.45)

### 3.2. Comparison of Occupational Grief Scores Based on Nurse Characteristics

The mean score of the nurses’ GSSN was 48.54 ± 9.458 (range: 22–84). A high level of occupational grief was observed in 265 pediatric emergency and ICU nurses. The analysis revealed statistically significant differences in occupational grief scores based on factors including age, religious beliefs, duration of work in the unit, death education training, education on the terminal phase and grief, and whether the nurses had experienced the loss of a close friend or family member (Table 2).

**TABLE 2:** Comparison of occupational grief scores among nurses with different characteristics.

Variable	*n* (%)	Total score of nurses’ grief, mean ± SD	Statistic	*p*
Gender				
Male	8 (3.02)	46.25 ± 7.40	*t* = −0.69	0.489
Female	257 (96.98)	48.61 ± 9.52		
Age				
< 31	150 (56.61)	46.59 ± 8.28	*F* = 9.57	< 0.01^∗^
31∼40	89 (33.58)	50.21 ± 9.49		
> 40	26 (9.81)	54.04 ± 12.43		
Level of Education				
College and below	9 (3.40)	52.11 ± 10.17	*F* = 0.67	0.511
Bachelor’s Degree	246 (92.83)	48.39 ± 9.40		
Graduate degree	10 (3.77)	48.80 ± 10.71		
Family residence				
Countryside	128 (48.30)	49.03 ± 9.71	*F* = 0.56	0.569
Town	52 (19.62)	48.77 ± 7.80		
City	85 (32.08)	47.65 ± 10.02		
Religious belief				
Yes	21 (7.92)	55.71 ± 15.90	*t* = 2.22	0.038^∗^
No	244 (92.08)	47.92 ± 8.46		
Civil status				
Married	130 (49.06)	48.45 ± 10.41	*F* = 0.01	0.990
Single	132 (49.81)	48.61 ± 8.53		
Divorced	3 (1.13)	48.67 ± 7.09		
Years of service in the unit				
≤ 3 years	90 (33.96)	46.69 ± 7.56	*F* = 3.86	0.022^∗^
4∼10 years	94 (35.47)	48.47 ± 8.64		
> 10 years	81 (30.57)	50.67 ± 11.67		
Education on death				
Yes	104 (39.25)	45.61 ± 8.95	*t* = −4.18	< 0.01^∗^
No	161 (60.75)	50.43 ± 9.32		
Education on terminal period and grief				
Yes	167 (63.02)	46.95 ± 7.00	*t* = −3.19	0.002^∗^
No	98 (36.98)	51.23 ± 12.16		
Loss of close family member or friend				
Yes	189 (71.32)	50.48 ± 8.35	*t* = 5.08	< 0.01^∗^
No	76 (28.68)	43.71 ± 10.34		
Number of patient deaths experienced				
1∼5	222 (83.77)	48.46 ± 9.31	*F* = 0.13	0.880
6∼10	10 (3.77)	47.80 ± 9.30		
> 10	33 (12.45)	49.24 ± 10.67		

*Note: t*: *t*‐test, *F*: ANOVA.

Abbreviation: SD, standard deviation.

^∗^ *p* < 0.05.

Specifically, the analysis revealed that occupational grief scores varied significantly across age groups (*F* = 9.57 and *p* < 0.01). Nurses aged over 40 years reported the highest grief scores (54.04 ± 12.43), followed by those aged 31–40 years (50.21 ± 9.49), while the youngest group (≤ 30 years) had the lowest scores (46.59 ± 8.28). This suggests that older nurses may experience more intense occupational grief, possibly due to longer exposure to patient loss and cumulative emotional burden. Nurses who reported having a religious belief scored significantly higher on occupational grief (55.71 ± 15.90) compared with those without (47.92 ± 8.46) (*t* = 2.22 and *p* = 0.038). This may reflect a more profound existential engagement with death and loss among religious individuals. Significant differences were found based on years of service (*F* = 3.86 and *p* = 0.022). Nurses with more than 10 years of service had the highest grief scores (50.67 ± 11.67), while those with ≤ 3 years had the lowest (46.69 ± 7.56). Those with 4–10 years of service scored intermediately (48.47 ± 8.64). This indicates that grief may accumulate with prolonged exposure to end‐of‐life care environments. Nurses who had received education on death had significantly lower grief scores (45.61 ± 8.95) than those who had not (50.43 ± 9.32) (*t* = −4.18, *p* < 0.01). This underscores the potential protective role of death education in mitigating occupational grief. Similarly, nurses who received specific education on the terminal phase and grief reported lower grief scores (46.95 ± 7.00) compared with those who did not (51.23 ± 12.16) (*t* = −3.19 and *p* = 0.002). This further supports the value of targeted educational interventions. Nurses who had experienced the loss of a close family member or friend had significantly higher grief scores (50.48 ± 8.35) than those who had not (43.71 ± 10.34) (*t* = 5.08 and *p* < 0.01). This highlights how personal bereavement may amplify occupational grief.

### 3.3. The Scoring Distribution and Correlation Analysis of Various Scales

The mean GSSN score was 48.54 ± 9.458 (range: 22–84), and the DGS score was 17.75 ± 5.443 (range: 7–35). The PGSS‐IN score was 90.332 ± 16.743 (range: 27–135). Additionally, the CD‐RISC‐10 score was 23.18 ± 6.919 (range: 0–40), and the REQ score was 10.97 ± 3.606 (range: 4–20).

Pearson correlation analysis revealed a negative correlation between the total occupational grief score and factors such as psychological resilience, psychological detachment, competence in handling patient deaths, acceptance of patient deaths, and psychological preparedness for patient deaths (*r* = −0.247, −0.184, −0.296, −0.421, and −0.351, respectively; all *p* < 0.05). A positive correlation was also found between the total occupational grief score and disenfranchised grief score among nurses (*r* = 0.317 and *p* < 0.05). The correlations among the variables are shown in Table 3.

**TABLE 3:** Pearson correlation results of the study variables (*N* = 265).

Variables		1	2	3	4	5	6	7
1. Occupational grief score	*r*	1						
	*p*							

2. Psychological resilience	*r*	−0.247^∗∗^	1					
	*p*	< 0.001						

3. Psychological detachment	*r*	−0.184^∗∗^	0.315^∗∗^	1				
	*p*	0.003	< 0.001					

4. Competence in handling patient deaths	*r*	−0.296^∗∗^	0.364^∗∗^	0.051	1			
	*p*	< 0.001	< 0.001	0.406				

5. Acceptance of patient deaths	*r*	−0.421^∗∗^	0.254^∗∗^	0.143^∗^	0.516^∗∗^	1		
	*p*	< 0.001	< 0.001	0.020	< 0.001			

6. Psychological preparedness for patient deaths	*r*	−0.351^∗∗^	0.236^∗∗^	0.081	0.487^∗∗^	0.593^∗∗^	1	
	*p*	< 0.001	< 0.001	0.186	< 0.001	< 0.001		

7. Disenfranchised grief	*r*	0.317^∗∗^	−0.099	−0.057	−0.084	−0.190^∗∗^	−0.184^∗∗^	1
	*p*	< 0.001	0.107	0.357	0.173	0.002	0.003	

^∗^ *p* < 0.05.

^∗∗^ *p* < 0.001.

### 3.4. Multiple Linear Regression Analysis of Factors Influencing Occupational Grief Among Nurses in This Study

The total occupational grief score among nurses was used as the dependent variable in the multivariate linear regression analysis. Six statistically significant variables from the univariate analysis (age, presence of religious belief, years of work experience, receipt of death education training, receipt of end‐of‐life care training, and experience of a close family member or friend’s death) and statistically significant variables from the correlational analysis (disenfranchised grief, psychological resilience, psychological detachment, competence in handling patient deaths, acceptance of patient deaths, and psychological preparedness for patient deaths) were included. The specific coding of these variables is presented in Table 4. Collinearity diagnostics indicated that all models had tolerance values < 1 and variance inflation factors < 5, suggesting the absence of multicollinearity among the independent variables. Multivariate linear regression analysis revealed that age, receipt of death education training, experience of a close family member or friend’s death, disenfranchised grief, and acceptance of patient deaths were significant factors influencing occupational grief among nursing graduate students (*p* < 0.05), accounting for 41.5% of the total variance. The detailed results are presented in Table 5.

**TABLE 4:** Argument assignment.

Independent variable	Assignment mode
Age	≤ 30 years = 1; 31–40 years = 2; > 41 years = 3
Religious belief	Yes = 0; No = 1
Years of service in the unit	≤ 3 years = 1; 4–10 years = 2; > 10 years = 3
Education on death	Yes = 0; No = 1
Education on terminal period and grief	Yes = 0; No = 1
Loss of close family member or friend	Yes = 0; No = 1
Disenfranchised grief	Original input
Resilience scale	Original input
Psychological detachment	Original input
Competence in handling patient deaths	1 = very incompetent, 2 = incompetent, 3 = average, 4 = competent, 5 = very competent
Acceptance of patient deaths	1 = very unacceptable, 2 = not acceptable, 3 = average, 4 = acceptable, 5 = very acceptable
Psychological preparedness for patient deaths	1 = none, 2 = insufficient, 3 = average, 4 = sufficient, 5 = very sufficient

**TABLE 5:** The results of the multivariate analysis.

	***B***	**SE**	**Beta’**	***t***	**p**	**95% CI**	

Age	4.469	1.912	0.141	2.337	0.020	0.703	8.235
Education on death	2.439	0.987	0.126	2.470	0.014	0.494	4.383
Loss of close family member or friend	−4.199	1.073	−0.201	−3.915	< 0.01	−6.312	−2.087
Disenfranchised grief	0.329	0.090	0.189	3.670	< 0.01	0.152	0.506
Acceptance of patient deaths	−2.730	0.847	−0.216	−3.223	0.001	−4.398	−1.062

## 4. Discussion

### 4.1. Occupational Grief Was Found to be at a High Level Among Pediatric Emergency and ICU Nurses in This Study

The mean occupational grief score among pediatric emergency and ICU nurses in this study was 48.54. Based on the original scale classification—Normal (≤ 48.45), High (48.46–58.85), and Severe (> 58.85)—this result indicates a high level of grief among the participating nurses. This level appears elevated when compared with previous studies that generally reported moderate grief levels among pediatric nurses [30, 31]. The high level of occupational grief observed may be understood through the lens of disenfranchised grief within a challenging socioprofessional context. In China, factors such as low social recognition of nursing, high workloads, and complex nurse–patient relationships, may collectively suppress the legitimate expression of professional mourning. When grief is not socially validated or adequately resolved, it tends to accumulate, potentially explaining the elevated scores we found compared with studies in Western settings. These findings underscore the need for greater focus on the psychological well‐being of nurses in pediatric emergency and ICU settings and highlight the importance of developing targeted interventions to mitigate occupational grief.

### 4.2. Occupational Grief Among Pediatric Emergency and ICU Nurses Is Influenced by Multiple Factors

This study found that older nurses with more extensive work experience in emergency departments and ICUs exhibited higher levels of occupational grief. Research indicates that prolonged work in the ICU increases exposure to patient loss [30]. Each additional loss, compounded by previous experiences, along with a heavy workload, can contribute to intense grieving among nurses.

Pediatric emergency and intensive unit care nurses who received death education training in this study reported lower levels of occupational grief. Similar to our findings, Rodriguez et al. [31] found that education on grief and death led to lower and less intense levels of occupational grief. Training on grief and death can help nurses better understand the dying process, improve confidence in care quality, reduce stress, and develop effective coping strategies, leading to less intense grieving. Current research shows that the nine‐module end‐of‐life care education curriculum by the American Association of Critical Care Nurses (AACNs) covers key areas such as principles of end‐of‐life nursing care, pain management, symptom management, ethical/legal issues, cultural considerations, communication, sorrow and mourning, attainment of end‐of‐life quality care, and preparation and care at the time of death [32]. This curriculum effectively enhances nurses’ care quality and their ability to manage grief and distress. Therefore, clinical leaders should offer educational courses on death or end‐of‐life care training to help nurses develop a proper perspective on life and death. This enables each nurse to confront occupational grief directly, armed with knowledge and the ability to seek help when necessary.

Our research shows that nurses experience heightened occupational grief when they have personally lost a close friend or family member. Studies indicate that nurses often experience occupational grief when interacting with terminally ill patients and their families, with grief manifesting similarly to personal bereavement but also exhibiting professional characteristics [33]. This may occur because, when nurses face patient death, they are reminded of their own experiences of losing a loved one, which intensifies their grief.

This study identified disenfranchised grief as a significant factor influencing occupational grief among pediatric emergency and ICU nurses (*B* = 0.329, *p* < 0.01). Nurses with more severe disenfranchised grief tended to experience higher levels of occupational grief. This may be because the bond between the child and the nurses in emergency and intensive care is not publicly recognized [34]. Medical professionals involved in nurturing relationships with children and their families may experience grief that they feel unable to express openly, as it is often considered less acceptable by their peers and community. This societal attitude compounds their emotional burden. Unresolved grief among healthcare workers can lead to the long‐term accumulation of grief, exacerbating the levels experienced by pediatric nurses [35]. Therefore, nursing managers should provide channels for nurses to express their grief, such as through prayer and the sharing of nursing memories. When a nurse experiences a patient death, nursing managers should arrange shifts flexibly to allow adequate rest and time for self‐care before resuming work.

This study found that acceptance of patient death significantly influenced occupational grief among pediatric emergency and ICU nurses (*B* = −2.730 and *p* = 0.001). Higher acceptance of patient death was associated with lower levels of occupational grief. Nurses with a positive attitude toward patient death tend to view dying and death more constructively, allowing them to confront these situations calmly. This higher level of self‐efficacy in coping with death contributes to lower levels of occupational grief [23, 36]. Additionally, these nurses may possess strong psychological preparedness and acceptance, recognizing that despite their best efforts, they may not be able to save their patient’s life, viewing it as a painful but ultimately liberating end for the patient. Therefore, it is recommended that nursing managers provide targeted psychological counseling for nurses experiencing occupational grief or facilitate support from psychology and psychiatry specialists [37–39]. Such supportive measures can help alleviate occupational grief, promote nurses’ psychological well‐being, and ultimately contribute to the stability and development of the pediatric nursing team.

The multivariate linear regression analysis revealed that neither psychological resilience nor psychological detachment significantly predicted occupational grief. Several interrelated factors may explain these findings. First, occupational grief in pediatric emergency and critical care settings is a complex, context‐dependent emotional response, likely influenced more by environmental and situational factors than by stable individual traits such as psychological resilience. Evidence suggests that interventions aimed at improving the work environment may be more effective in mitigating this grief than those focusing solely on individual attributes [40]. Second, psychological detachment may function primarily as a short‐term, culturally influenced coping strategy. While it can buffer immediate emotional distress, it may not substantially alleviate the underlying experience of occupational grief. Among Chinese nurses, cultural norms emphasizing emotional restraint and professional responsibility may further moderate the observable relationship between detachment and grief‐related outcomes. Therefore, psychological detachment appears to be a situational, culturally shaped behavior whose association with occupational grief is not linear, underscoring the need to consider specific cultural and contextual frameworks when evaluating its role [37, 41].

This study reveals that occupational grief among pediatric emergency and ICU nurses represents a significant psychological burden requiring systematic intervention. The identified factors—particularly disenfranchised grief, personal loss experiences, and limited death education—point to both individual and organizational dimensions of this challenge. For nursing leaders, these findings underscore the critical need to transform workplace culture from one that implicitly expects emotional suppression to one that explicitly supports grief processing. Specifically, healthcare institutions should integrate comprehensive death education into continuing professional development, establishing it as a core clinical competency rather than an optional training. Furthermore, the strong association between disenfranchised grief and occupational grief highlights the necessity of creating formal and informal channels for emotional expression, such as structured debriefing sessions and peer support programs. Clinical managers should also recognize that nurses’ personal bereavement histories significantly impact professional functioning, warranting tailored psychological support. Ultimately, addressing occupational grief requires a dual approach: enhancing individual coping resources through education while simultaneously reforming organizational practices to validate and accommodate the emotional labor of nursing. Such systemic changes are essential not only for preserving nurses′ mental health but also for ensuring the sustainability of high‐quality pediatric critical care services.

### 4.3. Advantages and Limitations

This study possesses several notable strengths. First, it addresses a significant gap in the literature by providing one of the first quantitative, cross‐sectional investigations into occupational grief among pediatric emergency and ICU nurses in China. While previous research has largely emerged from Western contexts, this study offers valuable insights within the unique sociocultural and healthcare environment of China, characterized by high workloads and complex nurse–patient relationships. Second, the use of multiple validated and reliable instruments, including the GSSN, the DGS, and scales measuring resilience, psychological detachment, and professional support, allows for a comprehensive and multidimensional assessment of the phenomenon. Furthermore, the inclusion of a substantial sample from 13 tertiary hospitals enhances the robustness of the findings and the application of multivariate analysis successfully identified key modifiable factors, such as death education and acceptance of patient death, providing a solid evidence base for targeted interventions.

However, several limitations must be acknowledged. The cross‐sectional design, while useful for establishing associations, precludes any determination of causality between the identified factors and occupational grief. The dynamic nature of grief suggests that longitudinal studies are needed to understand its trajectory over time. Second, the study utilized a convenience sampling method restricted to tertiary hospitals in Southern China, which may limit the generalizability of the findings to nurses in other regions, in primary or secondary care settings, or in different cultural contexts within China. Furthermore, the absence of stratified sampling may affect the representativeness of the results. Future studies could consider adopting random sampling combined with stratified sampling and increasing the sample size to enhance the representativeness of the sample. Data were collected through self‐report questionnaires, which are susceptible to social desirability bias and recall bias. Despite these limitations, the findings offer crucial preliminary evidence and a foundation for future research, including longitudinal studies, qualitative explorations of lived experiences, and the development of interventional strategies tailored to the needs of nurses in high‐acuity pediatric settings.

### 4.4. Implications for Nursing Management

This study provides critical insights into occupational grief among pediatric nurses. This study underscores the imperative for healthcare institutions to proactively address occupational grief through systematic interventions. Key managerial strategies should include integrating comprehensive death education into professional development, establishing accessible psychological support systems, and fostering a unit culture that validates emotional expression. By implementing structured debriefing sessions and ensuring adequate recovery time after patient deaths, nursing leaders can mitigate grief accumulation and preserve both staff wellbeing and care quality in these high‐acuity settings.

## 5. Conclusion

In conclusion, this study found that occupational grief among pediatric emergency and ICU nurses is at a high level. Various factors influence the intensity of occupational grief in this group, including age, receipt of death education, experience with the death of a close individual, the degree of disenfranchised grief, and acceptance of patient death. To address this issue, healthcare institution managers can mitigate nurses’ occupational grief and promote psychological well‐being through the following strategies: (1) Offering systematic death education or end‐of‐life care education programs. Regular educational programs should be provided to help pediatric emergency and ICU nurses develop a comprehensive understanding of life and death. These courses should focus on cultivating a nuanced perspective on mortality and end‐of‐life care. (2) Establishing a standardized psychological support system for nurses. For those working prolonged hours in pediatric emergency and ICUs, regular psychological counseling or mental health support should be made available. (3) Nursing administrators should encourage nurses to express their grief appropriately. Providing opportunities for nurses to express their emotions and offer support after a patient’s death is crucial to help them cope with their grief. Reasonable scheduling should also be implemented to ensure nurses have adequate recovery time before resuming work. Although this study highlights the occupational grief experienced by pediatric emergency and critical care unit nurses, it was limited to 13 hospitals in Southern China, which may not fully represent experiences in other regions of the country. Future research should explore occupational grief across different regions or among different populations to enhance the generalizability of the findings.

## Author Contributions

Yongqi Liang, Caixin Yin, Li Sun, Hui Liu, Jinlu Chen, Yan Zhou, Yi Luo, and Xuan Shi conducted the research design and collected data. Yongqi Liang, Xiaoke Jiang, and Yue Peng analyzed the data and wrote the manuscript. Xiaoke Jiang, Yue Peng, and Caixin Yin revised the manuscript.

## Funding

This study was supported by the Guangdong Nursing Association, Lingnan Nightingale Nursing Institute, Guangdong Province (YJYM202304).

## Disclosure

All authors approved the final manuscript.

## Ethics Statement

In this study, all principles of publication ethics and principles of the Declaration of Helsinki were respected. The study was approved by the Ethics Committee of Guangzhou Women and Children’s Medical Center, Guangzhou Medical University (Ethics Approval Number: [2023] No. 342A01). Ethical approval for this study was granted on December 27, 2023.

## Conflicts of Interest

The authors declare no conflicts of interest.

## Data Availability

The data that support the findings of this study are available on request from the corresponding author. The data are not publicly available due to privacy or ethical restrictions.
